# Use of the second-generation antipsychotic, risperidone, and secondary weight gain are associated with an altered gut microbiota in children

**DOI:** 10.1038/tp.2015.135

**Published:** 2015-10-06

**Authors:** S M Bahr, B C Tyler, N Wooldridge, B D Butcher, T L Burns, L M Teesch, C L Oltman, M A Azcarate-Peril, J R Kirby, C A Calarge

**Affiliations:** 1 Department of Microbiology, University of Iowa, Iowa City, IA, USA; 2 Department of Psychiatry, University of Iowa, Iowa City, IA, USA; 3 Department of Epidemiology, University of Iowa, Iowa City, IA, USA; 4 High Resolution Mass Spectrometry Facility, University of Iowa, Iowa City, IA, USA; 5 Department of Internal Medicine and Iowa City Veterans Affairs Health Care System, University of Iowa, Iowa City, IA, USA; 6 Department of Cell Biology and Physiology and Microbiome Core Facility, University of North Carolina, Chapel Hill, NC, USA; 7 Baylor College of Medicine, The Menninger Department of Psychiatry and Behavioral Sciences, Texas Children's Hospital, Houston, TX, USA

## Abstract

The atypical antipsychotic risperidone (RSP) is often associated with weight gain and cardiometabolic side effects. The mechanisms for these adverse events are poorly understood and, undoubtedly, multifactorial in etiology. In light of growing evidence implicating the gut microbiome in the host's energy regulation and in xenobiotic metabolism, we hypothesized that RSP treatment would be associated with changes in the gut microbiome in children and adolescents. Thus, the impact of chronic (>12 months) and short-term use of RSP on the gut microbiome of pediatric psychiatrically ill male participants was examined in a cross-sectional and prospective (up to 10 months) design, respectively. Chronic treatment with RSP was associated with an increase in body mass index (BMI) and a significantly lower ratio of Bacteroidetes:Firmicutes as compared with antipsychotic-naïve psychiatric controls (ratio=0.15 vs 1.24, respectively; *P*<0.05). Furthermore, a longitudinal observation, beginning shortly after onset of RSP treatment, revealed a gradual decrease in the Bacteroidetes:Firmicutes ratio over the ensuing months of treatment, in association with BMI gain. Lastly, metagenomic analyses were performed based on extrapolation from 16S ribosomal RNA data using the software package, Phylogenetic Investigation of Communities by Reconstruction of Unobserved States (PICRUSt). Those data indicate that gut microbiota dominating the RSP-treated participants are enriched for pathways that have been implicated in weight gain, such as short-chain fatty acid production.

## Introduction

Over the last two decades, the prescribing rate of second-generation antipsychotic medications (SGAs) to treat children has increased nearly eightfold, in light of evidence supporting their efficacy.^1, 2, 3^ However, significant weight gain and associated cardiometabolic disturbances are common side effects, placing patients at an elevated risk for type 2 diabetes and cardiovascular disease.^1, 4, 5, 6^ Alarmingly, the life expectancy of patients with severe mental illness is 20–25 years shorter than that of the general population, primarily due to increased cardiovascular disease.^7, 8, 9^

The mechanisms underlying SGA-induced weight gain are not well understood. The metabolic side effects are believed to be multifactorial in their etiology, including pharmacological, genetic, lifestyle and environmental contributors.^10, 11, 12^ Recent evidence also implicates the gut microbiome, which impacts various aspects of metabolism, including energy harvest and the development of obesity.^3, 13, 14, 15, 16, 17^ Davey *et al.* have shown that acute olanzapine treatment in rats resulted in significant weight gain and cardiometabolic dysfunction in association with changes in the gut microbiota.^1, 2, 3^ Importantly, mice lacking a microbiota do not gain as much weight compared with non-treated controls.^18^

The interaction between the gut microbiota and SGAs may well be bidirectional. In fact, gut bacteria utilize neurotransmitter signaling pathways targeted by antipsychotic medications.^19, 20, 21^ On the other hand, they have a substantial role in xenobiotic pharmacokinetics. For example, the cleavage of the benzisoxazole ring system of risperidone (RSP) is a process effected primarily by the intestinal microbiota, yielding alternative metabolites.^7^ Microbiota-mediated mechanisms such as these can contribute to the emergence of adverse drug events, including weight gain.^7, 8, 9, 22^

In contrast to olanzapine, whose use in adolescents has been discouraged by the Food and Drug Administration due to its propensity to cause significant weight gain,^10, 11, 12^ RSP is one of the most commonly prescribed SGAs, particularly in children and adolescents.^2, 3^ RSP is, nonetheless, associated with a moderately high potential to induce weight gain.^4^ Thus, we performed a cross-sectional examination of the impact of chronic RSP exposure in children and adolescents on gut bacterial composition and on the corresponding metabolic pathways. We further explored the longitudinal dynamics of the gut microbiota following the onset of RSP treatment. Our results indicate that RSP use is associated with alteration of the gut microbiome that is enriched for metabolic pathways that might cause or exacerbate weight gain.

## Materials and methods

### Sample collection

The cross-sectional study involved 18, 9–15-year-old, medically healthy males who had been on RSP for at least 1 year (chronic RSP group). The longitudinal study consisted of five males, aged 9–13 years, enrolled within 1 month of starting RSP. Ten 10–14-year-old participants, who were psychiatrically ill but not receiving any SGAs, served as controls. Height and weight were measured following standard guidelines during the research visits.^23^ Body mass index (BMI) was calculated and age–sex-specific BMI *Z*-scores were computed to account for natural growth.^24^ When available, anthropometric data were also extracted from the medical records to compute baseline BMI *Z*-score for RSP-treated participants within −31 to +3 days of RSP initiation.^23^ This allowed estimating the change in BMI *Z*-score between the time RSP was started and study visit. In order to do the same for the psychiatric control group, baseline BMI measurements were determined from the medical records to provide a comparable mean±s.d. interval between baseline BMI and BMI at time of stool sample collection across treatment groups. Stool samples were collected once in the cross-sectional study, and as frequently as every month in the longitudinal study. Stool samples were collected freshly and transported to the lab where they were aliquoted and frozen within 15–30 min. Alternatively, they were provided at home, placed on dry ice supplied by research staff and picked up within 24 h (while still frozen). Samples were stored at −80 °C until DNA extraction and were excluded from the study when antibiotic use occurred within 6 months of sample collection. The studies were approved by the Institutional Review Board at the University of Iowa.

### Multiplex DNA sequencing and assembly

Extracted genomic DNA was subjected to multiplex paired-end Illumina (Illumina, San Diego, CA, USA) sequencing of the V1-2 region of bacterial 16S ribosomal RNA (rRNA) genes with a MiSeq instrument (Chapel Hill, NC, USA) in the UNC Microbiome Core Facility. See Supplemental Methods for further details about the analysis.

## Results

Eighteen male participants, mean age 12.2 (s.d.=2.5) years, provided stool samples for the cross-sectional study, after a mean of 3.6 (s.d.=2.4) years of RSP treatment. In addition to RSP, participants also used psychostimulants (*n*=18, 100%), α-2 agonists (*n*=12, 66%) and selective serotonin reuptake inhibitors (*n*=2, 11%). Over the course of treatment, BMI *Z*-score increased by a mean of 0.31 (s.d.=1.11) points. Ten boys who had not received RSP served as psychiatric controls. Their mean age was 12.0 (s.d.=1.8) years. They were taking psychostimulants (*n*=7, 70%), α-2 agonists (*n*=3, 30%) and selective serotonin reuptake inhibitors (*n*=2, 20%). Over a comparable time interval (see Methods section), their BMI *Z*-score remained virtually unchanged (mean ΔBMI *Z*-score=0.09, s.d.=0.61). Psychiatric diagnoses and gastrointestinal symptoms were not different between the two groups, nor was food intake (Supplementary Table 1).

The longitudinal study consisted of five participants aged between 9 and 13 years (mean=11.7, s.d.=1.1), with a mean BMI *Z*-score at study entry of 0.12 (s.d.=0.84). Participants provided a stool sample within a few days (mean=3.2, s.d.=5.2) of starting RSP treatment and then up to monthly, for 10 months. Over the course of the treatment, BMI *Z*-scores increased by a mean of 0.28 (s.d.=0.23) units. All five participants were additionally taking psychostimulants and three of them (60%) were also taking α-2 agonists.

### Chronic RSP treatment is associated with a distinct gut microbiome

To evaluate whether microbial diversity is altered during chronic treatment with RSP, we surveyed fecal bacterial populations using 16S rRNA sequencing. The chronic RSP group displayed a significantly higher Shannon diversity when compared with psychiatric control participants (5.9 vs 5.2, respectively, *P*<0.05; Supplementary Figures 1A and B). There was a higher phylogenetic diversity (*P*=0.05; Supplementary Figures 1C and D) as well as a numerically, but not statistically, increased number of observed species in psychiatric control participants (Supplementary Figures 1E and F).

**Figure 1:**
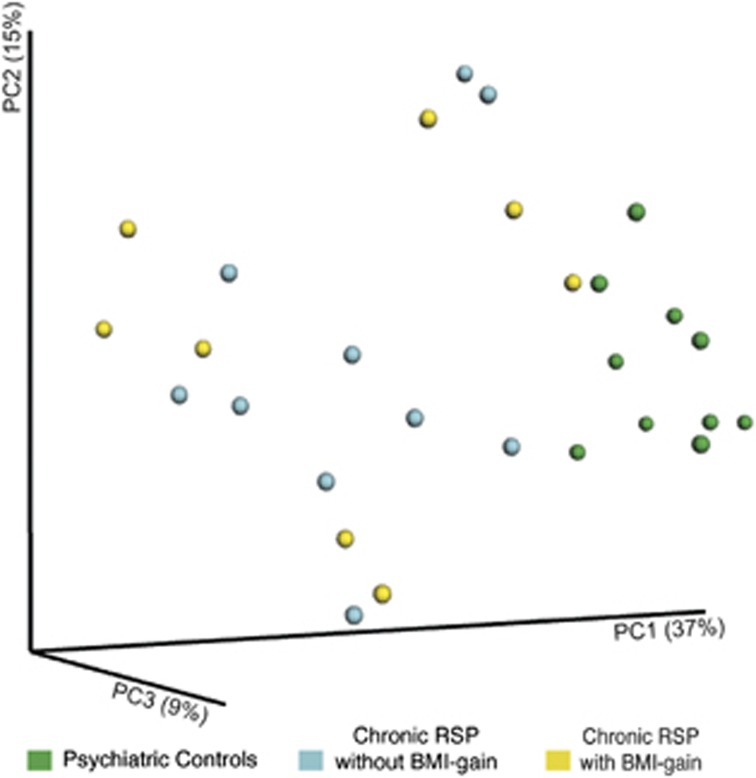
Differences in the fecal microbial communities in chronic risperidone (RSP)-treated participants vs psychiatric controls. PcoA of unweighted UniFrac distances between psychiatric control participants (green), chronic RSP-treated participants with significant body mass index (BMI) gain (blue, i.e., an increase in age−sex-specific BMI *Z*-score⩾0.5 units since starting RSP), and RSP-treated participants without significant BMI gain (yellow). Each point shows the average distance between individuals. Results are derived from bacterial V1−V2 16S rRNA data sets. ANOSIM *R*=0.5169, *P*=0.0001. PCoA, principal component analysis.

Using UniFrac, a phylogenetic distance metric, participants in the chronic RSP group were significantly different from psychiatric controls (analysis of similarity (ANOSIM) *R*=0.516, *P*<0.05). This indicates that a robust difference exists between the overall gut microbial profiles of the two groups (Figure 1). However, using this same metric among participants taking RSP, there was no significant difference (ANOSIM *R*=0.001, *P*=0.41) in microbial profiles between those who had a significant BMI gain (*n*=10), defined as a BMI *Z*-score increase by ⩾0.5 units, and those who did not (*n*=8; Supplementary Figure 2B).

### Chronic and acute treatment with RSP are associated with weight gain and alterations in gut bacterial composition

**Figure 2:**
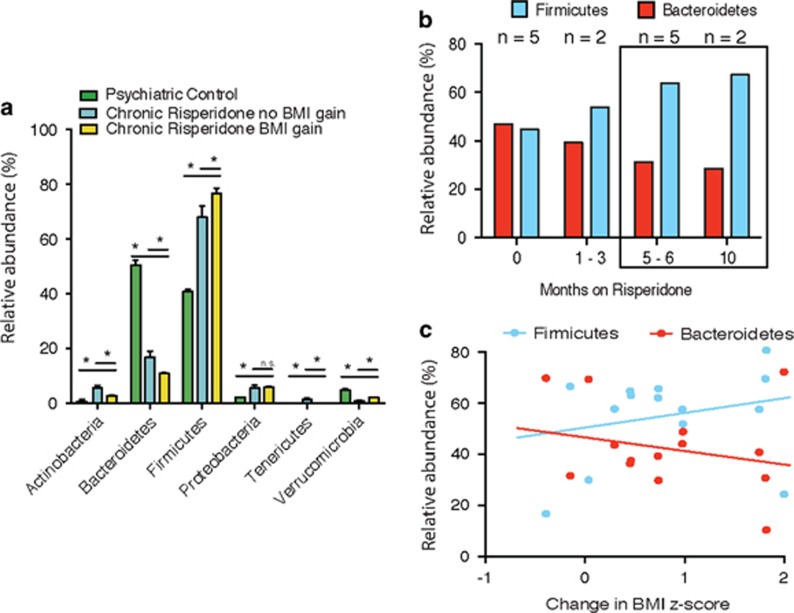
Phyla-level abundances. (**a**) Phyla-level abundances in psychiatric controls (green), chronic risperidone (RSP)-treated participants with significant body mass index (BMI) gain (blue, that is, an increase in age–sex-specific BMI *Z*-score ⩾0.5 units since starting RSP) and chronic RSP-treated participants without significant BMI gain (yellow). (**b**) Trajectory of change over time in the two major gut bacterial phyla, Bacteroidetes and Firmicutes, following the initiation of RSP. (**c**) Correlation of change in percent abundance of Bacteroidetes and Firmicutes, and change in BMI *Z*-score following the initiation of RSP.

The most common bacterial phyla in healthy adults are Firmicutes and Bacteroidetes, with significant representation of Actinobacteria and Proteobacteria. The relative proportions of these phyla sometimes diverge widely, reflecting not only interpersonal, geographical and lifestyle variations but also perturbations caused by disease.^25^ In our study, the psychiatric control participants displayed a distribution of the two major gut phyla, Firmicutes and Bacteroidetes, comparable to that observed in healthy populations^26^ (Figure 2a and Supplementary Table 2). In contrast, participants treated long-term with RSP and who also displayed a significant BMI gain had significantly less Bacteroidetes than Firmicutes relative to controls (ratio=0.20 vs 1.24, respectively, *P*<0.05). Importantly, significant differences in bacterial composition also differentiated RSP-treated participants who exhibited significant BMI gain from those who did not (Figure 2a and Supplementary Table 2). Finally, the bacterial composition within each of the two RSP treatment groups (regardless of changes in BMI *Z*-score) was different from that found in psychiatric controls (Figure 2a and Supplementary Table 2). Thus, both long-term RSP treatment as well as significant RSP-related BMI gain are independently associated with an altered distribution of the gut microbiota.

Data from sequential stool sampling revealed an increase in Firmicutes relative to Bacteroidetes, apparent within 1–3 months after initiating RSP treatment, that is, with the first follow-up stool sample (Figure 2b). The trend became more prominent over a longer period of exposure. Importantly, the percent abundance of Firmicutes and Bacteroidetes appeared to correlate with the RSP-induced increase in BMI *Z*-score, albeit not significantly, likely owing to the small sample size (for Bacteroidetes: Spearman's *r*=−0.29, *P*=0.30; for Firmicutes: *r*=0.32, *P*=0.26; Figure 2c).

### Discriminatory OTUs are detectable within treatment groups associated with weight gain

**Figure 3:**
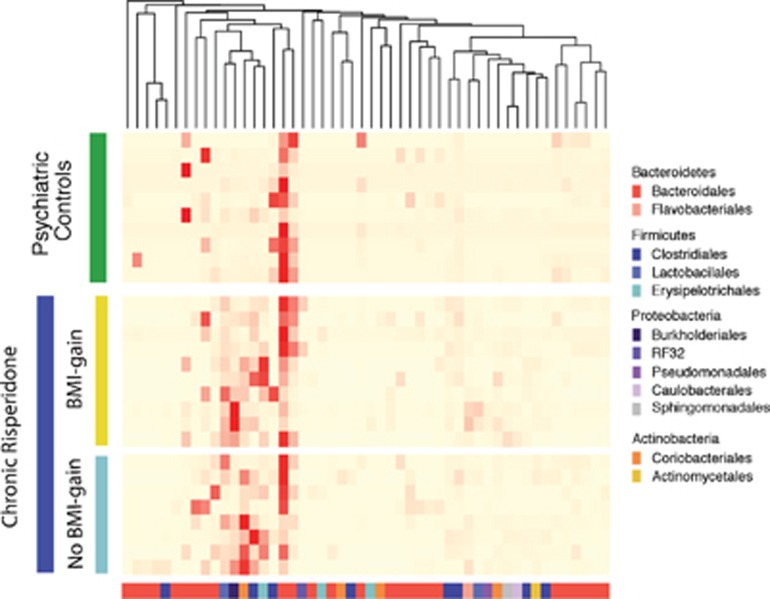
Relative abundance of unique genus-level operational taxonomic units (OTUs) of the gut microbiota of chronic risperidone (RSP)-treated participants and psychiatric controls. Fifty unique OTUs were defined by three methods (Metastats comparison, random forests algorithm and linear discriminant analysis effect size analysis). Genus-level OTUs are colored by phyla (bottom, x-axis) and individual biological replicates (left, green or blue), where red hues denote increased relative abundance of a unique OTU. BMI, body mass index.

Fifty key operational taxonomic units (OTUs) were identified as sufficient to discriminate between psychiatric controls and chronic RSP participants (Figure 3). Only three genera, belonging to the phylum Bacteroidetes, were more abundant in the psychiatric controls. In contrast, 47 OTUs were significantly more prevalent in the RSP participants, the most abundant being *Clostridium* sp., *Collinsella aerofaciens, Lactobacillus* sp.*, Ralstonia* sp. and the Erysipelotrichaceae family.

Importantly, *Clostridium* sp.*, Lactobacillus* sp.*, Ralstonia* sp. and Erysipelotrichaceae family members were more abundant in participants in the chronic RSP group who also displayed a significant BMI gain. In contrast, the order Coriobacteriales, specifically *C. aerofaciens,* was more abundant in participants within the chronic RSP group who did not show a significant BMI gain. These results suggest that specific Firmicutes and Burkholderiales species may have a role in RSP-induced weight gain while Coriobacteriales may be protective. In addition, Ruminococcaceae family members were most relatively abundant in RSP participants with significant BMI gain compared with psychiatric controls, whereas *Bacteroidetes* spp. were most relatively abundant in psychiatric controls compared with RSP participants regardless of BMI gain (Supplementary Figure 3).

### Gene frequency reveals altered microbial metabolic functionality following chronic treatment with RSP

**Figure 4:**
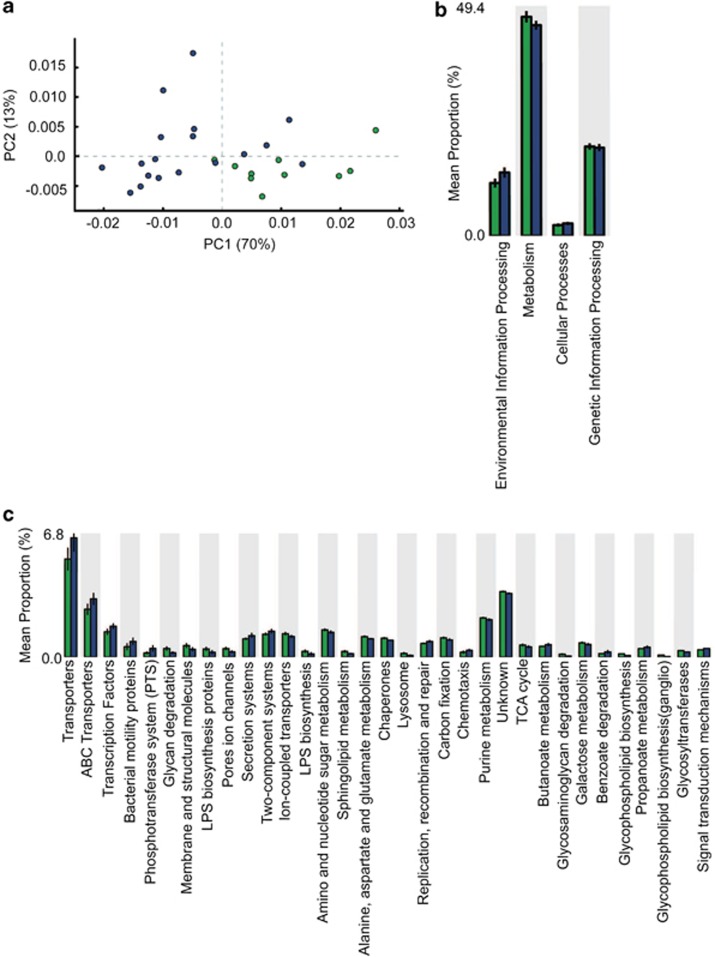
Phylogenetic Investigation of Communities by Reconstruction of Unobserved States (PICRUSt) predicted KEGG (Kyoto Encyclopedia of Genes and Gene Systems) orthologs. 1212 Significant KEGG orthologs were predicted with PICRUSt software. (**a**) PCoA of KEGG orthologs in chronic risperidone (RSP)-treated participants vs psychiatric controls. (**b**, **c**) Global and individual KEGG pathways of chronic RSP-treated participants (blue) vs psychiatric controls (green). PCoA, principal component analysis.

To predict the potential consequences of altering microbial gut composition in response to RSP treatment, we assessed the metabolic capacity of the microbiota based on the presence of organisms discerned by 16S rRNA sequences utilizing the algorithm, Phylogenetic Investigation of Communities by Reconstruction of Unobserved States (PICRUSt).^27^ Overall, out of 6909 potential metabolic features, we found 1212 KEGG (Kyoto Encyclopedia of Genes and Gene Systems) orthologs that were significantly different between the two groups (*P*<0.05; Figure 4). Families of KEGG orthologs were found to vary based on treatment group using principal component analysis (Figure 4a). By assigning KEGG orthologs to specific bacterial metabolic pathways, it was revealed that participants in the chronic RSP treatment group had microorganisms that were enriched for environmental information processing pathways (for example, response to antibiotics) and cellular processes (Figure 4b). In contrast, psychiatric control participants had significantly more orthologs classified in bacterial metabolism pathways (Figure 4b). A more detailed analysis revealed that those pathways prominent in the chronic RSP treatment group included membrane transport, cell motility, xenobiotics biodegradation and metabolism, transcription and signal transduction (Figure 4c). Within the xenobiotics biodegradation and metabolism category, major pathways included butanoate, propanoate, fatty acid and tryptophan metabolism (Figure 4c), all of which have been implicated in weight gain.^28, 29^ Conversely, pathways in greater abundance in psychiatric controls included glycan biosynthesis and metabolism, as well as metabolism of cofactors and vitamins. Here, glycan biosynthesis comprises mostly lipopolysaccharide biosynthesis, which is found primarily in Gram-negative organisms such as *Bacteroidetes* spp. The latter were vastly decreased in the RSP participants. Thus, PICRUSt reveals a potential mechanistic link between the observed shifts in bacterial taxa and their corresponding metabolic potential in response to treatment with RSP.

## Discussion

To our knowledge, this is the first description of a shift in the human gut microbiota following the use of SGAs. We found a significant shift in Bacteroidetes and Firmicutes in participants exposed chronically to RSP (76 and 11%, respectively) when compared with psychiatric controls (51 and 41%, respectively). The magnitude of these differences was most prominent in those participants who had gained significant weight. These differences in the gut microbiome composition following chronic treatment are consistent with findings from the prospective study where similar changes appeared to correlate with the magnitude of change in BMI *Z*-score.

The long-term health sequelae of obesity are significant and include cardiovascular disease and type 2 diabetes,^30^ particularly when obesity develops in childhood. Pediatric patients suffering from psychiatric disorders are at an elevated risk for obesity due to disease-related factors; this was a primary reason for selecting as controls participants with psychiatric illness but who are SGA naive. Although pharmacotherapy is often unavoidable to address serious symptoms and improve functioning, it is concerning that SGAs induce weight gain, compounding the health risks associated with obesity. SGA-induced weight gain is multifactorial and it is likely that the gut microbiome is a key contributor. In fact, a 3-week treatment with olanzapine induced weight gain in rats and led to a 4.3% decrease in Bacteroidetes.^1^ Despite the differences between the rat and human microbiomes, it is notable that, similar to olanzapine-treated rats, our participants treated chronically with RSP exhibited significantly lower relative prevalence of Bacteroidetes. Notably, a decrease in Bacteroidetes of a comparable magnitude has been reported in obese individuals, although the evidence is not without inconsistency.^13, 31^ Importantly, our results from the longitudinal study provide preliminary confirmation that a change in gut bacterial composition (including a decrease in Bacteroidetes) follows the initiation of RSP treatment and correlates with weight gain during the initial treatment phase.

Using Unifrac, which is an index of overall bacterial profile, we found no significant association between those who exhibited significant BMI gain and those who did not, following RSP treatment (Figure 1). However, when individual phyla, genera and species were compared, chronic RSP participants with a significant BMI gain were found to harbor a unique set of organisms, which distinguishes them from the chronic RSP participants without BMI gain (Figure 2). Therefore, it would be critical to examine how baseline microbial profile may shape one's propensity to gain weight following the initiation of SGAs.

To identify specific taxa that correlate with significant weight gain following RSP treatment, we described discriminatory OTUs associated with each treatment group (Figure 3). For psychiatric controls, the discriminatory OTUs were attributable to the class Bacteroidales. In contrast, for participants in the chronic RSP group with significant BMI gain, *Ralstonia* sp.*, Clostridium* sp. and members of the Erysipelotrichaceae family were the most abundant OTUs. In contrast, *C. aerofaciens* was the most abundant discriminatory OTU in the chronic RSP group without significant BMI gain. Taken together, these results suggest that alterations in the membership of Firmicutes and Actinobacteria phyla may drive overall major changes in the gut microbiota in relation to long-term RSP treatment. In contrast, alterations in the membership of Firmicutes and Proteobacteria may be linked to weight gain following long-term RSP treatment. This raises the possibility for Actinobacteria to be used for the development of novel therapeutics (prebiotics or probiotics) to prevent or reverse weight gain following RSP treatment. Indeed, it has been shown that Bacteriodales species not only ameliorate gut abnormalities (often found in individuals with psychiatric disorders) but may also restore social and behavioral impairments.^32, 33, 34^

Gut bacterial metabolic pathways have also been linked to obesity.^13, 35^ In fact, bacterial fermentation of complex carbohydrates and proteins produce short-chain fatty acids in the proximal colon, optimizing caloric harvest from the diet.^13^ Short-chain fatty acids directly provide energy to colonocytes, and absorption into the portal circulation stimulates adipogenesis.^19, 28^ In addition, short-chain fatty acids are believed to control anorectic hormone (peptide YY and glucagon-like peptide-1) release or production through the free fatty acid receptor 2 and free fatty acid receptor 3.^36^ In our study, higher levels of KEGG-associated pathways for butyrate and propionate metabolism were found within the RSP treatment group compared with psychiatric controls (Figure 4), suggesting that the gut microbiome of these individuals may have higher levels of short-chain fatty acids production, potentially leading to weight gain.

Our analysis using PICRUSt also found that the microbiota of participants treated chronically with RSP were enriched for KEGG orthologs affecting tryptophan metabolism (Figure 4). Tryptophan and its metabolite, 5-hydroxytryptamine or serotonin, are both critical to a number of physiological processes, including affective regulation and energy homeostasis. Growing evidence has also implicated neurotransmitters in bacterial signaling, further supporting the vast connectivity of the microbiome–gut–brain axis.^20, 37^ Specifically, gut bacteria are essential to the normal development of peripheral and central serotonin signaling pathways in the host.^38^ Moreover, probiotic administration has been shown to influence the availability of tryptophan, suggesting that microbiota manipulation could be a valid therapeutic strategy to modulate central nervous system signaling.^39^ In light of our findings, we speculate that alterations in tryptophan metabolism in the gut may mediate, at least in part, SGA-induced weight gain. Altered tryptophan metabolism may also affect the therapeutic benefit of RSP treatment.

This study has several limitations that must be acknowledged. First, the sample size of both studies was relatively small. This may have led to false-positive results. However, in light of the preclinical findings reviewed earlier and the apparent consistency in the pattern of change in bacterial composition across both human studies (that is, decreased Bacteroidetes:Firmicutes ratio), it is likely that our findings reflect a true effect of RSP treatment. It may be possible that changes in bacterial composition reflected an earlier change in dietary intake induced by RSP. However, the dietary data collected identified no differences between the groups. Polypharmacy, which is often observed in SGA-treated children, may additionally have a role in altering the microbiome. However, neither psychostimulant nor selective serotonin reuptake inhibitor use was associated with changes in gut bacterial composition in our participants (Supplementary Figure 2). Nonetheless, our findings require replication in a larger cohort, including females and in the absence of polypharmacy. Moreover, as diet has a pivotal role in shaping the gut microbiome, an evaluation of the impact of the western diet on RSP-induced weight gain will be critical for future studies to undertake.

Taken together, our findings offer preliminary evidence that the human gut microbiome is altered in patients treated chronically with RSP and may be associated with weight gain. Significant alterations in the microbiome were seen at the phyla level following both acute and chronic exposure to RSP. In addition, discriminatory genera (OTUs) were identified, which may help inform efforts to develop probiotics as a therapeutic. Prospective studies in both humans and mice are underway to further investigate the basis of these findings.
