# Supplementary figures and images for: Use of the second-generation antipsychotic, risperidone, and secondary weight gain are associated with an altered gut microbiota in children
Source: Transl Psychiatry. 2015 Oct 6;5(10):e652–. doi: 10.1038/tp.2015.135 (PMC4930121; doi:10.1038/tp.2015.135)

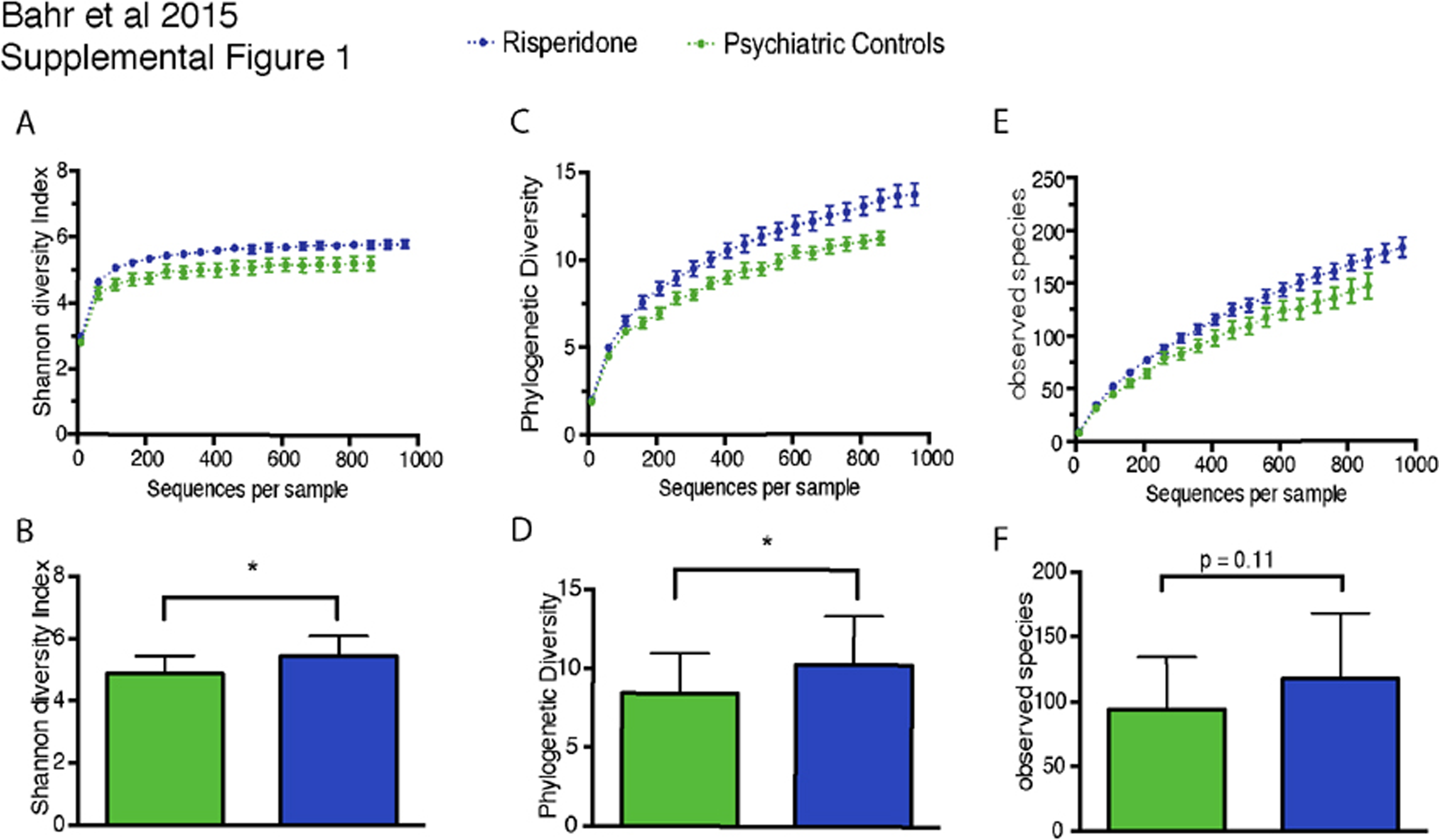

Supplement: Supplementary Figure 1 [file tp2015135x1.tif]

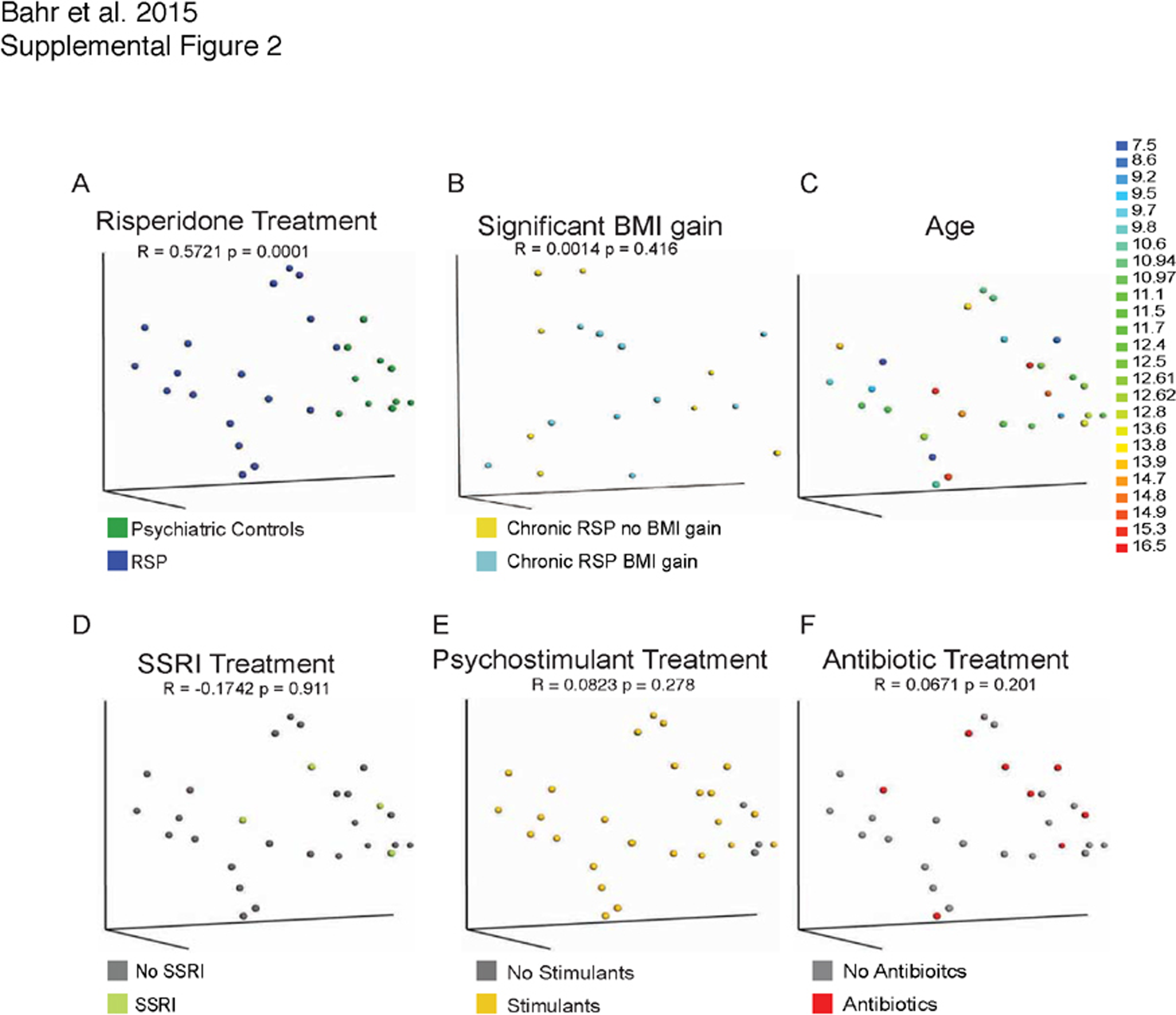

Supplement: Supplementary Figure 2 [file tp2015135x2.tif]

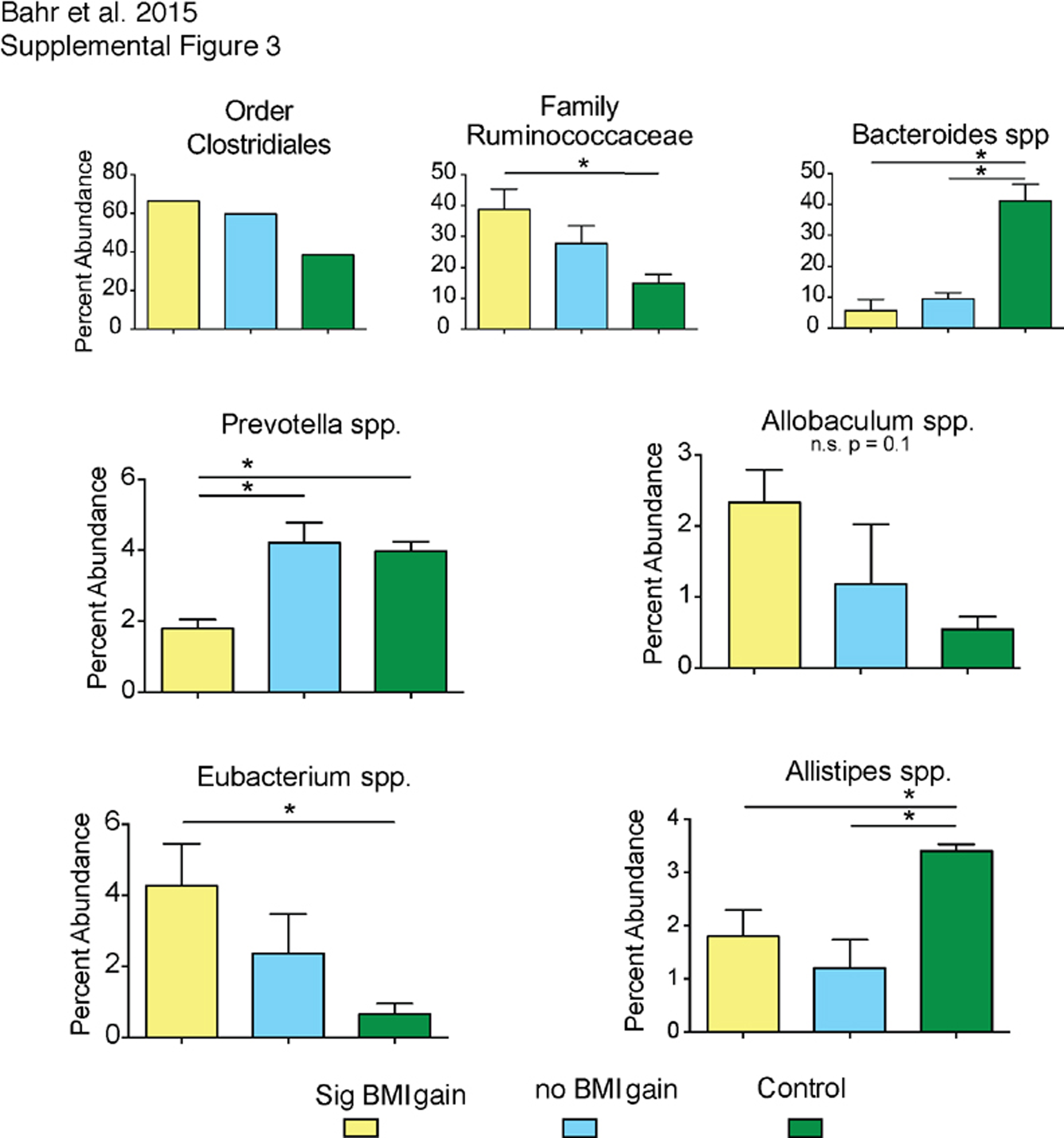

Supplement: Supplementary Figure 3 [file tp2015135x3.tif]

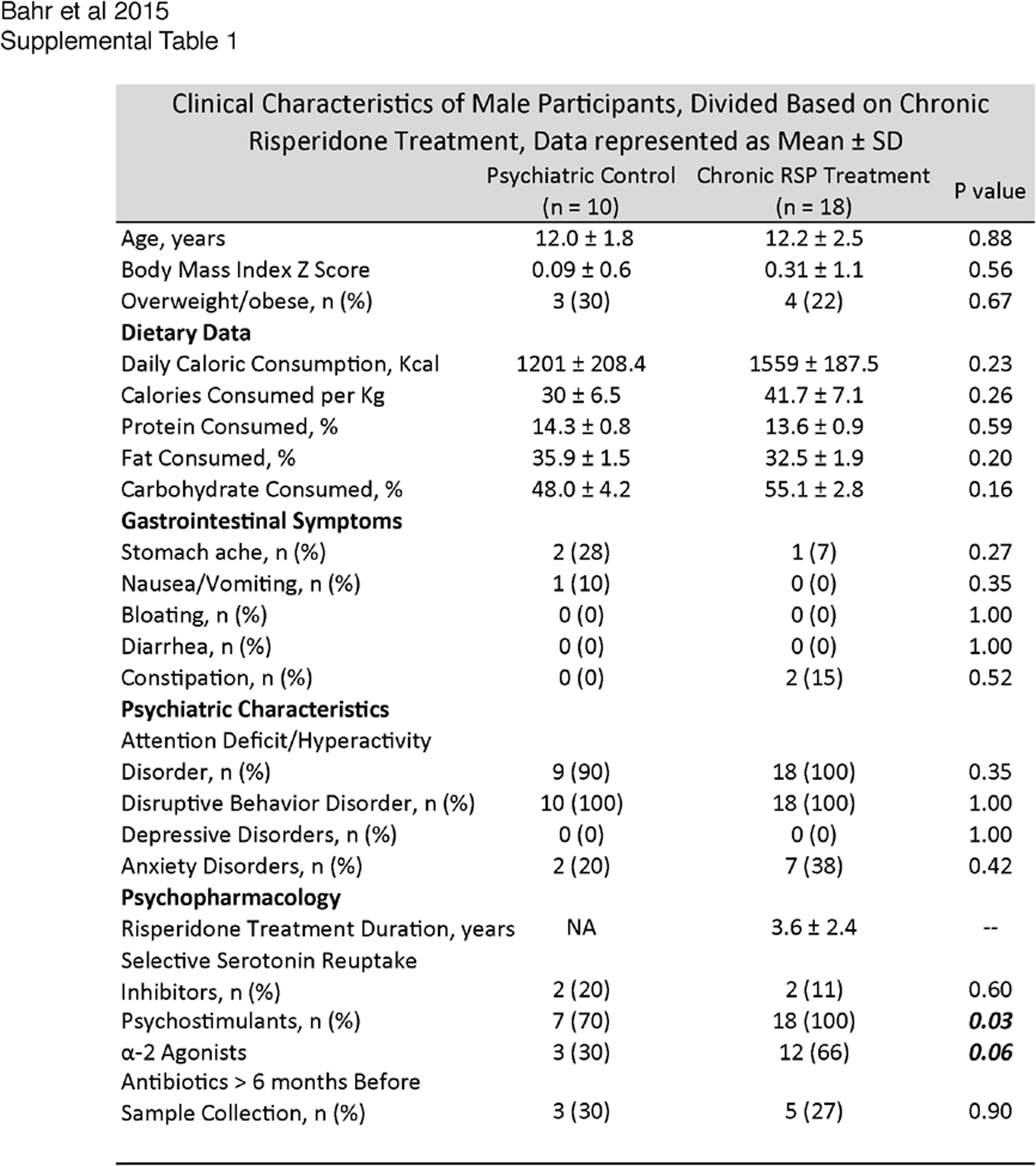

Supplement: Supplementary Table 1 [file tp2015135x4.tif]

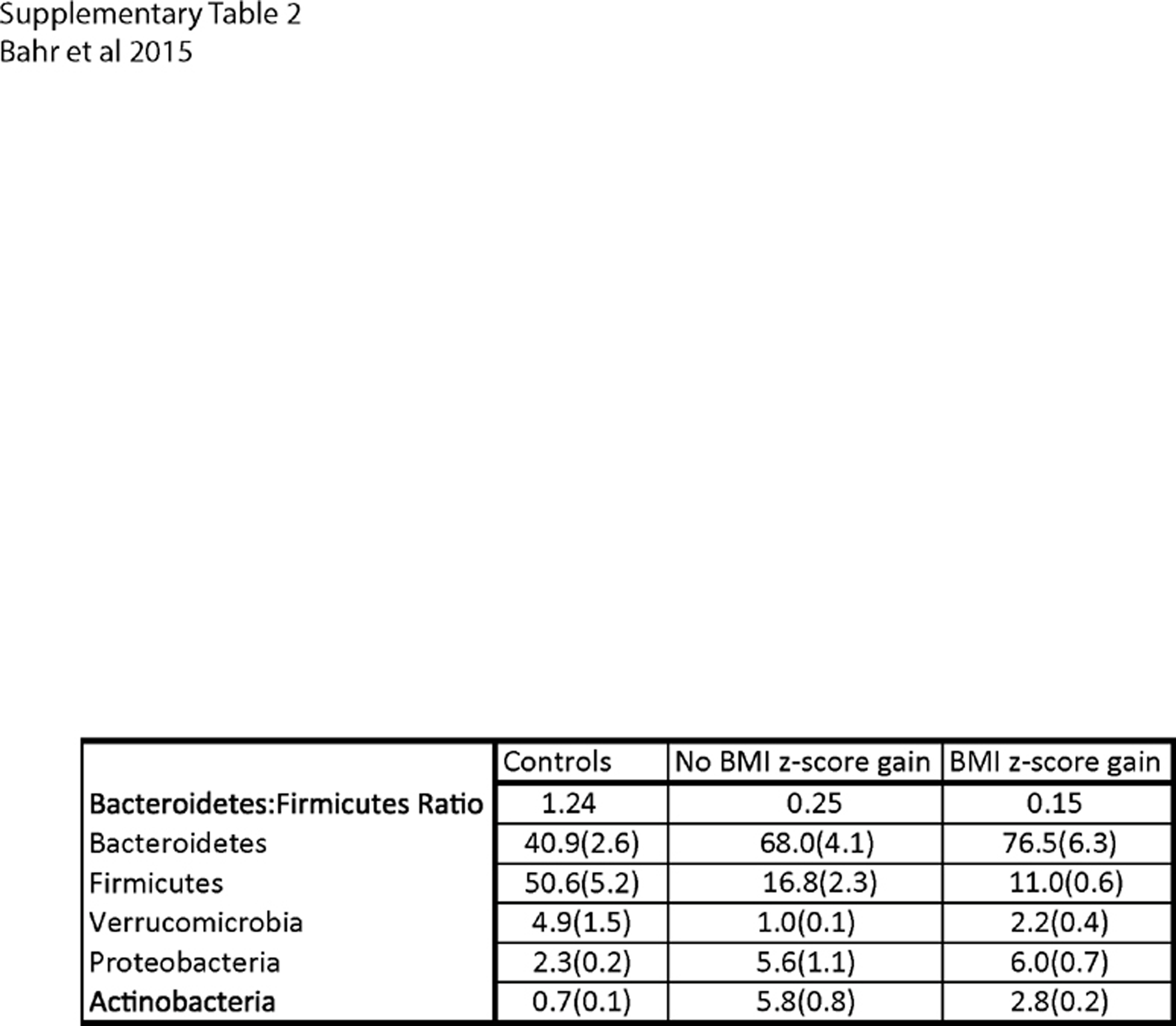

Supplement: Supplementary Table 2 [file tp2015135x5.tif]
