# Supplementary material for: Use of the second-generation antipsychotic, risperidone, and secondary weight gain are associated with an altered gut microbiota in children
Source: Transl Psychiatry. 2015 Oct 6;5(10):e652–. doi: 10.1038/tp.2015.135 (PMC4930121; doi:10.1038/tp.2015.135)
Supplement: Supplementary Information [file tp2015135x6.doc]

Supplementary Figure 1. **Shannon diversity index of the gut microbiota in chronic RSP-treated participants vs. psychiatric controls.** 1,000 iterations result in psychiatric controls (green) presenting with significantly lower overall diversity compared to chronic RSP-treated participants (blue).

Supplementary Figure 2. **Differences in the fecal microbial communities of chronic RSP-treated participants vs. psychiatric controls.** A-F) PCoA of unweighted UniFrac distances illustrating the association between gut bacterial clustering and various predictors of interest. Each point shows the average distance between an individual’s microbiome. Results are derived from bacterial V4 16S rRNA datasets. A) Chronic RSP-treated participants (blue) vs. psychiatric controls (green), R = 0.5169 p = 0.0001. B) Chronic RSP-treated participants with significant BMI gain (blue i.e., an increase in age-sex-specific body mass index (BMI) Z-score ≥ 0.5 units since starting risperidone) or without (yellow), R = 0.0014 p = 0.416. C) Age, youngest (blue) to eldest (red) D) SSRI-treated (chartreuse) vs. SSRI-untreated participants (grey), R = -0.1017 p = 0.775. E) Psychostimulants-treated (yellow) vs. Psychostimulants-untreated (grey), R = 0.1021 p = 0.241. F) Participants treated with antibiotics between 6 to 12 months prior to stool sample collection (red) vs. Participants not exposed to antibiotics for at least 12 months prior to stool sample collection (grey), R = 0.0932 p = 0.1702.

Supplementary Figure 3. **Relative abundances of discriminatory OTUs in RSP-treated participants vs. psychiatric controls.** A-C) The most highly abundant OTUs found to be discriminatory in Figure 3. Percent relative abundances are shown to be divided by psychiatric controls (green), RSP-treated participants with significant BMI-gain (yellow), and RSP treated participants without significant BMI-gain (blue). D-G) Low abundant OTUs found to be discriminatory in Figure 3.
